# Efficacy of rabies vaccines in dogs and cats and protection in a mouse model against European bat lyssavirus type 2

**DOI:** 10.1186/s13028-017-0332-x

**Published:** 2017-10-02

**Authors:** Tiina Nokireki, Miia Jakava-Viljanen, Anna-Maija Virtala, Liisa Sihvonen

**Affiliations:** 1 Finnish Food Safety Authority Evira, Mustialankatu 3, 00790 Helsinki, Finland; 2 Department of Veterinary Biosciences, Faculty of Veterinary Medicine, University of Helsinki, P.O. Box 66, 00014 Helsinki, Finland

**Keywords:** Antibody, EBLV-2, Lyssavirus, Protection, Rabies, Vaccination

## Abstract

**Background:**

Rabies is preventable by pre- and/or post-exposure prophylaxis consisting of series of rabies vaccinations and in some cases the use of immunoglobulins. The success of vaccination can be estimated either by measuring virus neutralising antibodies or by challenge experiment. Vaccines based on rabies virus offer cross-protection against other lyssaviruses closely related to rabies virus. The aim was to assess the success of rabies vaccination measured by the antibody response in dogs (n = 10,071) and cats (n = 722), as well as to investigate the factors influencing the response to vaccination when animals failed to reach a rabies antibody titre of ≥ 0.5 IU/ml. Another aim was to assess the level of protection afforded by a commercial veterinary rabies vaccine against intracerebral challenge in mice with European bat lyssavirus type 2 (EBLV-2) and classical rabies virus (RABV), and to compare this with the protection offered by a vaccine for humans.

**Results:**

A significantly higher proportion of dogs (10.7%, 95% confidence interval CI 10.1–11.3) than cats (3.5%; 95% CI 2.3–5.0) had a vaccination antibody titre of < 0.5 IU/ml. In dogs, vaccination with certain vaccines, vaccination over 6 months prior the time of antibody determination and vaccination of dogs with a size of > 60 cm or larger resulted in a higher risk of failing to reach an antibody level of at least 0.5 IU/ml. When challenged with EBLV-2 and RABV, 80 and 100% of mice vaccinated with the veterinary rabies vaccine survived, respectively. When mice were vaccinated with the human rabies vaccine and challenged with EBLV-2, 75–80% survived, depending on the booster. All vaccinated mice developed sufficient to high titres of virus-neutralising antibodies (VNA) against RABV 21–22 days post-vaccination, ranging from 0.5 to 128 IU/ml. However, there was significant difference between antibody titres after vaccinating once in comparison to vaccinating twice (P < 0.05).

**Conclusions:**

There was a significant difference between dogs and cats in their ability to reach a post vaccination antibody titre of ≥ 0.5 IU/ml. Mice vaccinated with RABV-based rabies vaccines were partly cross-protected against EBLV-2, but there was no clear correlation between VNA titres and cross-protection against EBLV-2. Measurement of the RABV VNA titre can only be seen as a partial tool to estimate the cross-protection against other lyssaviruses. Booster vaccination is recommended for dogs and cats if exposed to infected bats.

## Background

Rabies is a fatal encephalomyelitis caused by lyssaviruses. Rabies virus (RABV) causes about 99% of all rabies cases in humans, mostly in Asia and Africa. Even though the case fatality is almost 100%, the disease can be prevented by pre-exposure vaccination and by post-exposure treatment shortly after exposure. Pre-exposure vaccination can prevent the disease, but booster vaccination upon exposure is required to ensure that the immune system responds optimally [1]. Thirteen other lyssavirus species have been accepted by the International Committee on Taxonomy of Viruses, and two additional species have been identified. Phylogenetic analysis and the virus–host relationship suggest that all 16 currently identified species of lyssaviruses can be divided into phylogroups. Phylogroup I comprises RABV, Duvenhage virus, European bat lyssavirus types 1 and 2 (EBLV-2), Australian bat lyssavirus, Aravan virus, Khujand virus, Irkut virus and Bokeloh bat lyssavirus, whereas Lagos bat virus, Mokola virus and Shimoni bat virus form phylogroup II [2, 3]. West Caucasian bat virus, Ikoma lyssavirus and Lleida bat lyssavirus may be representatives of possible new phylogroups [4–6]. Gannoruwa bat lyssavirus probably belongs to phylogroup I [7].

Human infections usually result from the bite of a rabid dog infected with RABV [1]. However, in a small number of cases, the infection results from contact with bats [8]. Two confirmed human cases caused by European bat lyssavirus type 2 (EBLV-2), one in Finland in 1985 [9, 10] and one in the UK in 2002 [11] have been reported. EBLV-2 is known to associate with two closely related *Myotis* bat species, Daubenton’s (*Myotis daubentonii*) and pond bats (*M. dascyneme*). EBLV-2 has sporadically been isolated from Daubenton’s bats in the Netherlands [12], Switzerland [13], the UK [14], Finland [15, 16], Germany [17], Denmark [18] and Norway [19]. Finland was declared a rabies-free country by the OIE in 1991. Nevertheless, rabies can remain a residual risk to public health due to the natural circulation of EBLV-2 [15, 16]. Spillovers from chiropters to carnivores are not unusual, and at least two spillovers of RABV from bats to carnivore hosts have occurred, which have succeeded in being maintained through time [20, 21]. No spillover infections to other mammals than humans have been detected for EBLV-2. In experimental infections, EBLV-2 has been shown to cause fatal disease in foxes [22], sheep [23] and mice [24, 25]. European bat lyssavirus type 1 (EBLV-1), however, has been detected in natural infections of stone martens (*Martes foina*) [26], sheep [27] and domestic cats [28].

The assessment of rabies vaccine efficacy [1, 29, 30] is usually undertaken by immunisation and virus challenge using laboratory animals (potency test). The efficacy of vaccination can be indirectly assessed by measuring antibodies from serum. Cellular immunity, however, is also important in clearing a virus infection [31]. Virus-neutralising antibody assays are used to verify that an immune response has occurred. An internationally accepted threshold titre of 0.5 IU/ml has been adopted [1]. However, it has been shown that vaccinated animals with low antibody levels have survived RABV challenge, indicating that other immune mechanisms also protect against rabies infection. On the other hand, according to Aubert [32], the presence of detectable neutralising antibodies against rabies at the time of challenge does not indicate protection for all animals. Nevertheless, animals with neutralising antibodies at the time of challenge are significantly better protected against RABV than those without detectable virus neutralizing antibodies (VNA) [32, 33]. Information on the protection and cross-protection efficacy of rabies vaccines against all other lyssaviruses under field conditions is still limited. Protection is inversely related to the genetic distance of the lyssavirus from RABV [34, 35].

The type of vaccine used, number of vaccinations, interval between vaccination and blood sampling, age at vaccination, sex, reproductive status, size and breed can all influence the antibody response of animals vaccinated against rabies [36–39]. In post-vaccination serological studies, the percentage of animals with inadequate titres has been 3.1–8.1% for dogs [36, 38, 40] and 2.85% for cats [36]. As many as 53% (95% CI 41–65%) of imported rescue dogs from Eastern Europe have been found to have inadequate titres after rabies vaccination [41].

Pre-exposure vaccination is recommended for people potentially at risk of rabies, such as veterinarians, laboratory workers and bat handlers. People who work with rabies viruses in laboratories or vaccination production facilities should have VNA tested every 6 months [1]. According to WHO recommendations, 0.5 IU/ml is a sufficient antibody level, considered to be proof of an adequate vaccination response, and a booster vaccination is recommended for levels lower than this. A series of booster vaccinations is also required after exposure to lyssavirus [1]. The same threshold titre is applied in dogs and cats to confirm a satisfactory response to vaccination prior to international travel. All vaccines currently available are based on RABV. According to studies conducted to investigate the cross-neutralisation of sera and cross-protection in a mouse model against different lyssaviruses, they offer variable cross-protection against other lyssaviruses [34, 35, 42–46]. However, there are no official recommendations by WHO concerning whether the current procedure of pre-and post-exposure treatment would need to be modified for people who are exposed to other lyssaviruses than RABV.

The aim of this study was to assess the success of rabies vaccination measured by the antibody response in dogs and cats, as well as to study the factors influencing the inability of animals to produce a sufficient antibody response. In addition, the aim was to assess how well an RABV-based rabies vaccine, selected based on the ability to induce an antibody response in dogs and cats, affords protection in a mouse model against intracerebral challenge with EBLV-2 isolated from a Finnish bat (FI-EBLV-2), as well as against challenge with RABV isolated from a Finnish raccoon dog (FIRD-RABV). We also examined the protection offered by a rabies vaccine for humans and the correlation between VNA titres and cross-protection against EBLV-2 in mice.

## Methods

### Viruses

A Finnish EBLV-2 (FI-EBLV-2) isolate obtained from a Daubenton’s bat in 2009 (GenBank Accession Number JX129233) was used as the challenge virus. The original bat brain suspension was intracerebrally inoculated into eight newborn mice (ScaNmri suckling mice) with each mouse receiving 20 µl of the suspension. The brain suspension from these eight newborn mice was subsequently inoculated into 4-week-old mice to amplify the virus and obtain the required quantity of virus stock to be used as the challenge virus in this study. RABV isolated from a Finnish raccoon dog (FIRD-RABV) (R1470/88) in 1988 was also used in this study. The original archived brain passage had been kept at − 70 °C since 1990, and for this challenge study it was grown in the brain of 4-week-old mice to amplify the virus and obtain the required quantity of virus stock.

### Vaccination and virus challenge of mice

Three- to four-week-old NMRI mice (Harlan, NL; n = 20 per challenge virus and n = 5 vaccine only) were vaccinated intra-peritoneally with 0.1 ml of vaccine diluted 1:10 in physiological saline solution with a 16-mm needle. When the human vaccine (Rabies-Imovax^®^; Sanofi-Pasteur MSD, France, batch G1391-4) was used, the mice were vaccinated either once (group a) or twice (group b) with a 2-week interval between the initial and the booster vaccination. When the animal vaccine (Rabisin^®^ vet; Merial, France, batch L374051) was used, the mice were vaccinated once. Vaccines were purchased from Helsinki University Pharmacy. The protocol was modified from the European Pharmacopoiea protocol [29]: the minimum lethal dose (MLD_50_) for the intra-cranial challenge was determined according to protocol of European Pharmacopoeia [29] and 50 MLD_50_ was used. The mice were challenged intra-cranially with 30 µl of virus suspension according to the procedure described by WHO [47] 28 days after vaccination. The mice were anesthetized by inhalation anaesthesia using isoflurane, and they were given 0.05–0.1 mg/kg buprenorphine hydrochloride subcutaneously at the time of intra-cranial challenge. The back titration of five mice per group was set up with 50 MLD_50_, 5 MLD_50_ and 0.5 MLD_50_ of each virus. Five mice per vaccine were not challenged. The mice were monitored twice per day for any clinical signs of rabies, and to minimize suffering they were killed when signs of rabies infection were obvious (weight loss, behavioural changes, neurological signs and paralysis) or when the observation period of 6 weeks had ended.

Serum was collected from the vaccinated mice prior to the challenge 21 or 22 days after the vaccination. The serum was collected from the lateral tail vein by appropriately trained personnel. The tail was first warmed and blood was collected with a 23-gauge needle. The target volume was 100 µl. At the end of the trial, the mice were euthanized either by the time they were showing clinical signs or after the monitoring period. To confirm the presence of rabies infection, the brains of the mice were collected and a fluorescent antibody test (FAT) [30] was performed on the brain. Smears prepared from a sample of brain tissue were fixed in high-grade cold acetone, air dried and then stained with specific conjugate (FITC Anti-Rabies Monoclonal Globulin, Fujirebio Diagnostics and Rabies Antinucleocapsid conjugate, Bio-Rad). FAT slides were then examined for specific fluorescence using a fluorescence microscope. The seroconversion of the vaccinated mice was analysed using the rapid fluorescent focus inhibition test RFFIT [30]. Five microlitres of sample was diluted 1:10. Serial dilutions of test sera were mixed with the challenge virus (CVS-11, ATCC VR-959) preparation and BHK-21 cells. Samples were fixed and stained with specific conjugate (FITC Anti-Rabies Monoclonal Globulin, Fujirebio Diagnostics). Residual virus was detected using a standard fluorescence microscope. The serum neutralisation end-point titre was defined as the dilution factor of the highest serum dilution at which 50% of the observed microscopic fields contained one or more infected cells. OIE/WHO human reference serum was used to convert the end point titre into IU/ml.

### Serological analysis of dogs and cats

The antibody responses of dogs and cats were determined using the FAVN test [48]. This test involves the neutralisation of a constant amount of rabies virus CVS-11 strain adapted to cell culture before inoculating cells susceptible to rabies virus (BHK-21 C13). The serum titre was the dilution at which 100% of the virus was neutralised in 50% of the wells. This titre was expressed in IU/ml by comparing it with the neutralising dilution of OIE serum of dog origin under the same test conditions.

### Selection of dog and cat samples

This was a case–control study with a duration of 5 years. During 2009–2013, 10,793 serum samples from dogs (n = 10 071) and cats (n = 722) were sent to the Finnish Food Safety Authority Evira for post-vaccination efficacy tests. Of these samples, 1055 dogs that had an antibody titre of < 0.5 IU/ml and for which submission data were available comprised the case group for dogs. An approximately similar number of dogs with submission data that had an antibody titre of ≥ 0.5 IU/ml were randomly assigned to the control group (n = 1626). In cats, only 25 had an antibody titre of < 0.5 IU/ml (cases), and a much larger number of cats that had antibody titre of ≥ 0.5 IU/ml were randomly assigned to the control group (n = 241). Submission forms for these samples were evaluated. Three inactivated rabies vaccines were used for dogs and cats in Finland during 2009–2013: Wistar-G52 strain vaccine, BHK-21cell vaccine with Pasteur RIV strain and Flury LEP strain vaccine.

### Statistical analysis

The 95% confidence intervals (CI) for valid percentages (excluding missing values) were calculated with Jeffrey’s method [49] using EpiTools [50]. Statistical analyses were performed using the statistical software SPSS 22.0 (IBM SPSS Statistical Package version 22, USA). The outcome variable was failure to reach the required antibody level (0 for antibody level ≥ 0.5 IU/ml, denoting the ability to reach the required antibody level, and 1 for < 0.5 IU/ml, denoting failure). Independent variables collated in the dataset were the vaccine used, age at vaccination, breed and size (only for dogs), gender, the number of vaccinations and the time from vaccination to sampling. Dogs were categorised into five different breed size groups based on their height (< 30 cm, 30–45 cm, 46–60 cm, > 60 cm and unknown). Furthermore, 277 different dog breeds were originally categorized into 10 groups by the Fédération Cynologique Internationale. Animals were divided into two age groups: up to 1 year and over 1 year old. Based on the time interval between vaccination and sampling, three groups were created: sampling less than 3 months after vaccination, 3–6 months after vaccination and more than 6 months after vaccination. First, Fisher’s exact test and crude logistic regression analyses were performed to examine the pairwise associations between the outcome and each independent variable separately. Variables with P ≤ 0.2 were included in the multivariable logistic regression analysis, with separate models for cats and dogs, and variables with Wald’s P < 0.05 were included in the final model. Correlations between independent variables were calculated with the Phi test (no important ones were found). A causal diagram was used to assess potential confounders; their impacts on the other variables in the model were verified, but none needed to be included in the models. Pairwise interactions were assessed. Since a significant interaction was found in dogs between the vaccine used and the age of the dog, two separate models were created: one for dogs up to 1 year old and another for older dogs. Additionally, for younger dogs, the time interval between vaccination and sampling was categorized into two groups: up to or more than 6 months. The goodness of fit of the final model was assessed with the Omnibus test, Nagelkerke’s R^2^ and the Hosmer and Lemeshow test, and by calculating the area under the curve (AUC).

## Results

### Results of the vaccine potency test

**Fig. 1:**
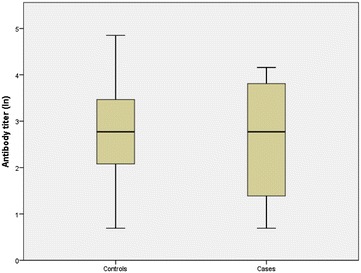
Comparison of VNA titers (ln) of the mice that survived the challenge (controls) and of the mice that succumbed after the challenge with EBLV-2 (cases). The interquartile range is the difference between the 75th and 25th percentiles and corresponds to the length of the box. The lines in the boxes represent the medians, the whiskers represent the minimum and maximum, unless there are values more than 1.5 times the interquartile range above 75th percentile, in which case it is the third quartile plus 1.5 times the interquartile range

**Fig. 2:**
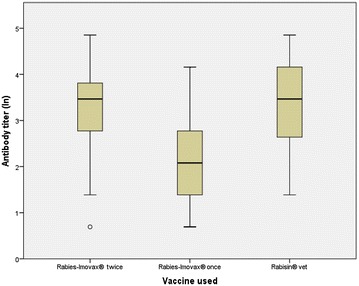
Comparison of VNA titers (ln) in three groups of mice vaccinated with different vaccines or vaccination schemes. The interquartile range is the difference between the 75th and 25th percentiles and corresponds to the length of the box. The lines in the boxes represent the medians, the whiskers represent the minimum and maximum, unless there are values more than 1.5 times the interquartile range above 75th percentile, in which case it is the third quartile plus 1.5 times the interquartile range. Circles represent outliers that are > 2 box lengths below the 25th percentile

When challenged with FIRD-RABV, all of the vaccinated mice survived. When challenged with FI-EBLV-2, 80% of the vaccinated mice survived after vaccination with the veterinary vaccine. When mice were vaccinated with the human vaccine, 75–80% of the mice survived, depending on the booster (Table 1). All vaccinated mice developed sufficient to high VNA titres against RABV, ranging from 0.5 to 128 IU/ml. The signs of rabies appeared 7–8 days post-infection with EBLV-2 and 6–7 days post-infection with RABV. Mice that succumbed after challenge with FI-EBLV-2 virus had a VNA titre of 2–64 IU/ml against RABV (individual results not shown). There were no statistically significant differences between the VNA titres of the mice that survived the challenge in comparison to those that succumbed after the challenge with EBLV-2 (Fig. 1). Mice vaccinated twice with Rabies-Imovax^®^ or once with Rabisin^®^ vet had significantly higher antibody titres than those vaccinated once with Rabies-Imovax^®^ (P < 0.05) (Fig. 2).

**Table 1:** Rabies vaccination protection following an intra-cranial challenge in mice

	Total number of mice	Survival after challenge with	
		FIRD-RABV n (%)	FI-EBLV-2 n (%)
Rabies Imovax, vaccinated twice	20	20 (100)	16 (80)
P value^1^		0.000	0.002
Rabies Imovax, vaccinated once	20	ND	15 (75)
P value^1^			0.005
Virus control 50 MLD_50_	5	0 (0)	0 (0)
Virus control 5 MLD_50_	5	2 (40)	2 (40)
Virus control 0.5 MLD_50_	5	4 (80)	4 (80)
Rabisin, vaccinated once	20	20 (100)	16 (80)
P value^1^			0.002
Virus control 50 MLD_50_	5	0 (0)	0 (0)
Virus control 5 MLD_50_	5	2 (40)	2 (40)
Virus control 0.5 MLD_50_	5	3 (60)	4 (80)

^1^P value derived using Fisher’s exact test for the number of vaccinated and challenged mice that survived relative to the total and compared with the 50 MLD_50_ virus control mice

### Results of the serological analysis of vaccinated dogs and cats

Of the 10,071 dog samples analysed during 2009–2013, 1073 (10.7%; 95% confidence interval CI 10.1–11.3) had a VNA titre of < 0.5 IU/ml. Of the 722 cats analysed, only 25 (3.5%; 95% CI 2.3–5.0) had a VNA titre of < 0.5 IU/ml.

### Risk factors for failure to produce sufficient antibody levels in dogs and cats

Descriptive data for the dogs and cats in the case–control study are provided in Tables 2 and 3. Younger dogs were associated with lower antibody titres (P < 0.05, data not shown). Since there was a significant interaction between the variables dog age and the vaccine used, two multivariable logistic regression models for dogs were constructed: one for dogs up to 1 year old and another for older dogs. For younger dogs, the Flury LEP vaccine was used significantly more often than for older dogs (P < 0.05, data not shown). For both age groups, dogs vaccinated with the Flury LEP vaccine only and dogs vaccinated with the Pasteur RIV vaccine only had a significantly higher risk of failing to reach an antibody level of 0.5 IU/ml in comparison to dogs vaccinated with the Wistar-G52 vaccine only (Tables 4, 5). Additionally, for both age groups, vaccination with the Flury LEP vaccine only had the highest risk of failing to reach an antibody level of 0.5 IU/ml. In younger dogs, these risks were higher than in older dogs. In younger dogs, if the time between vaccination and sampling was over 3 months, the risk of failing to reach an antibody level of 0.5 IU/ml was significantly higher than if the time span was shorter. In older dogs, the risk was higher if the time was over 6 months. For older dogs (over 1 year) of a larger size (> 30 cm), the risk of failing to reach an antibody level of 0.5 IU/ml was greater. In younger dogs, those over 60 cm had a higher risk compared to smaller dogs.

**Table 2:** Descriptive data on samples included in the statistical analysis of dog sera (n = 2681) tested for rabies antibody response after vaccination in Finland during 2009–2013

Variable	Levels	Antibody response < 0.5 IU/ml (n = 1055)		Antibody response ≥ 0.5 IU/ml (n = 1626)	
		n	% (95% CI)	n	% (95% CI)
Age at vaccination (year)					
≤ 1		544	52.2 (49.2–55.2)	328	20.3 (18.4–22.3)
> 1		498	47.8 (44.8–50.8)	1289	79.3 (77.7–81.6)
Gender					
Female		500	54.7 (51.5–57.9)	750	52.7 (50.1–55.3)
Male		414	45.3 (42.1–48.5)	672	47.3 (44.7–49.9)
Number of vaccinations					
Once		941	89.2 (87.2–91.0)	1071	65.9 (63.5–68.1)
Twice		99	9.4 (7.7–11.3)	433	26.6 (24.5–28.8)
More than twice		15	1.4 (0.8–2.3)	122	7.5 (6.3–8.9)
Time btw vaccination and sampling (months)					
< 3		21	2.3 (1.5–3.4)	158	11.1 (9.6–12.9)
3–6		335	36.8 (33.7–39.9)	459	32.3 (30.0–34.8)
> 6		555	60.9 (57.7–64.1)	802	56.5 (53.9–59.1)
Vaccine used					
Only vaccine Wistar-G52		53	7.3 (5.6–9.4)	229	22.1 (19.7–24.7)
Only vaccine Pasteur RIV		298	41.3 (37.7–44.9)	387	37.4 (34.5–40.4)
Only vaccine Flury LEP		315	43.6 (40.0–47.3)	233	22.5 (20.0–25.1)
Other		56	7.8 (6.0–9.9)	186	18.0 (15.7–20.4)
FCI group					
FCI1		142	15.0 (12.8–17.4)	186	13.4 (11.7–15.2)
FCI2		114	12.1 (10.1–14.2)	146	10.5 (9.0–12.2)
FCI3		106	11.2 (9.3–13.3)	191	13.7 (12.0–15.6)
FCI4		33	3.5 (2.5–4.8)	47	3.4 (2.5–4.4)
FCI5		138	14.6 (12.4–16.9)	183	13.2 (11.5–15.0)
FCI6		43	4.5 (3.4–6.0)	46	3.3 (2.5–4.3)
FCI7		75	7.9 (6.3–9.8)	77	5.5 (4.4–6.8)
FCI8		133	14.1 (12.0–16.4)	158	11.4 (9.8–13.1)
FCI9		109	11.5 (9.6–13.7)	266	19.1 (17.1–21.3)
FCI10		31	3.3 (2.3–4.6)	36	2.6 (1.8–3.5)
No FCI group		22	2.3 (1.5–3.4)	55	4.0 (3.0–5.1)
Breed size (cm)					
< 30		178	19.2 (16.8–21.9)	434	32.3 (29.9–34.9)
30–45		295	31.9 (28.9–34.9)	408	20.4 (28.0–32.9)
46–60		208	22.5 (19.9–25.2)	267	19.9 (17.8–22.1)
> 60		245	26.5 (23.7–29.4)	233	17.4 (15.4–19.5)

FCI group 1: Sheepdogs and Cattledogs (except Swiss Cattledogs), FCI group 2: Pinscher and Schnauzer—Molossoid and Swiss Mountain and Cattledogs, FCI group 3: Terriers, FCI group 4: Dachshunds, FCI group 5: Spitz and primitive types, FCI group 6: Scent hounds and related breeds, FCI group 7: Pointing Dogs, FCI group 8: Retrievers—Flushing Dogs—Water Dogs, FCI group 9: Companion and Toy Dogs, FCI group 10: Sighthounds

*CI* confidence interval, *n* number of cases

**Table 3:** Descriptive data on samples included in the statistical analysis of cat sera (n = 266) tested for the rabies antibody response after vaccination in Finland during 2009–2013

Variable	Levels	Antibody response < 0.5 IU/ml (n = 25)		Antibody response ≥ 0.5 IU/ml (n = 241)	
		n	% (95% CI)	n	% (95% CI)
Age at vaccination (year)					
≤ 1		12	48.0 (29.5–66.9)	47	19.6 (14.9–25.0)
> 1		13	52.0 (33.1–70.5)	193	80.4 (75.0–85.1)
Gender					
Female		5	20.0 (8.1–38.4)	89	58.6 (50.6–66.2)
Male		8	32.0 (16.4–51.5)	63	26.1 (20.9–31.9)
Number of vaccinations					
Once		24	96.0 (82.8–99.6)	194	80.5 (75.5–85.5)
Twice		1	4.0 (0.4–17.2)	42	17.4 (13.0–22.6)
More than twice		0	0.0 (0–9.5)	5	2.1 (0.8–4.5)
Time btw vaccination and sampling (months)					
< 3		1	5.3 (0.6–22.1)	78	42.2 (35.2–49.4)
3–6		8	42.1 (22.3–64.1)	70	37.8 (31.1–45.0)
> 6		10	52.6 (31.2–73.4)	37	20.0 (14.7–26.2)
Vaccine used					
Only vaccine Wistar-G52		3	25.0 (7.6–52.9)	39	25.7 (19.2–33.0)
Only vaccine Pasteur RIV		1	8.3 (0.9–32.8)	63	41.4 (33.8–49.4)
Only vaccine Flury LEP		6	50.0 (24.3–75.7)	25	16.4 (11.2–22.9)
Other		2	16.7 (3.6–43.6)	25	16.4 (11.2–22.9)

*CI* confidence interval, *n* number of cases

**Table 4:** Multivariable logistic regression results of risk factors for not reaching the antibody level of 0.5 IU/ml after vaccination against rabies in dogs up to 1 year old (n = 872) in Finland during 2009–2013

Independent variable	Logistic regression coefficient B	Wald’s *P* value	Odds ratio (95% CI)
Vaccine			
Wistar-G52 only	Na	Na	1
Pasteur RIV only	2.049	0.000	7.8 (4.1–14.5)
Flury LEP only	2.608	0.000	13.6 (7.0–26.2)
D other	0.466	0.406	1.6 (0.5–4.8)
Breed size (cm)			
< 30	Na	Na	1
30–60	0.308	0.221	1.4 (0.8–2.2)
> 60	1.159	0.000	3.2 (1.7–5.9)
Time btw vaccination and sampling (months)			
< 3	Na	Na	1
3–6	1.400	0.001	4.1 (1.8–9.1)
> 6	1.508	0.000	4.5 (2.0–10.4)
Constant	−2.907	0.000	0.055

*Na* not applicable, *CI* confidence interval, *other* dogs vaccinated abroad with vaccines not available in Finland or with a combination of different vaccines

**Table 5:** Multivariable logistic regression results of risk factors for not reaching the antibody level of 0.5 IU/ml after vaccination against rabies in dogs older than 1 year (n = 1787) in Finland during 2009–2013

Independent variable	Logistic regression coefficient B	Wald’s P value	Odds ratio (95% CI)
Vaccine			
Wistar-G52 only	Na	Na	1
Pasteur RIV only	0.926	0.000	2.5 (1.5–4.1)
Flury LEP only	1.271	0.000	3.6 (2.2–5.9)
Other	0.243	0.418	1.3 (0.7–2.3)
Breed size (cm)			
< 30	Na	Na	1
30–60	0.464	0.013	1.6 (1.1–2.3)
> 60	0.823	0.000	2.3 (1.5–3.5)
Time btw vaccination and sampling (months)			
< 6	Na	Na	1
> 6	1.432	0.000	4.2 (2.8–6.3)
Constant	−3.180	0.000	0.042

*Na* not applicable, *CI* confidence interval, *other* dogs vaccinated abroad with vaccines not available in Finland or with a combination of different vaccines

In cats, we observed no statistically significant differences between the vaccines used (Table 6). However, there was a similar tendency towards a higher risk of failing to reach an antibody level of 0.5 IU/ml for vaccination with the Flury LEP vaccine only compared to vaccination with the Wistar-G52 vaccine only. Cats that were vaccinated at the age of up to 1 year old had a significantly higher risk of failing to reach an antibody level of 0.5 IU/ml than cats vaccinated at an older age. Similarly to dogs, cats that were sampled for testing 3–6 months or over 6 months after vaccination had a significantly higher risk of failing to reach an antibody level of 0.5 IU/ml than cats that had been sampled less than 3 months after vaccination.

**Table 6:** Crude and multivariable logistics regression results of risk factors for not reaching the antibody level of 0.5 IU/ml after vaccination against rabies in cats in Finland during 2009–2013

Independent variable	Logistic regression coefficient B	Wald’s P value	Odds ratio (95% CI)
In crude logistic regressions			
Vaccine (n = 164)			
Wistar-G52 only	Na	1	Na
Pasteur RIV only	−1.578	0.178	0.2 (0.0–2.1)
Flury LEP only	1.138	0.130	3.1 (0.7–13.6)
Other	0.039	0.967	1.0 (0.2–6.7)
Age at vaccination (n = 265)			
< 1 year vs ≥ 1 year	1.332	0.002	3.8 (1.6–8.9)
Time btw vaccination and sampling (n = 204) (months)			
< 3	Na	1	Na
3–6	2.188	0.042	8.9 (1.1–73.1)
> 6	3.048	0.004	21.1 (2.6–170.9)
In multivariable logistic regression (n = 159)			
Vaccine			
Wistar-G52 only	Na	1	Na
Pasteur RIV only	−1.090	0.378	0.3 (0.03–3.8)
Flury LEP only	1.475	0.085	4.4 (0.8–23.5)
Other	0.827	0.439	2.3 (0.3–16.6)
Age at vaccination			
< 1 year vs ≥ 1 year	2.282	0.006	9.8 (1.9–48.8)
Time btw vaccination and sampling (months)			
< 3	Na	1	Na
3–6	1.615	0.19	5.0 (0.5–56.2)
> 6	2.906	0.026	18.3 (1.4–235.3)
Constant	−5.439	0.000	0.004

*Na* not applicable, *CI* confidence interval, *other* cats vaccinated abroad with vaccines not available in Finland or with a combination of different vaccines

## Discussion

New lyssaviruses related to RABV have been discovered, and it is possible that there may still be undetected bat lyssaviruses in many parts of the world. Bats do not often interact with people, but transmission of lyssaviruses to humans and pets has been documented. Finland experienced a human death from EBLV-2 in 1985 [9, 10], and better knowledge of the effectiveness of cross-protection is therefore needed to predict the impact of rabies vaccination if exposed to infected bats, as EBLV-2 appears to be enzootic at least in some areas in Finland [15, 16]. The immune response elicited by RABV-based rabies vaccines has been shown to be capable of cross-protection against those lyssaviruses in phylogroup I, but not for those that do not belong to this phylogroup [42–45]. However, even though EBLV-2 belongs to the same phylogroup I as RABV, the protection induced by rabies vaccines has only been limited in an experimental virus challenge study in mice, even with the production of VNAs [34].

VNAs are the main method of protection during rabies infection, and the role of cell-mediated and innate immunity is poorly understood. Measuring the VNA titre is currently the most common way to assess the success of vaccination, and an internationally accepted threshold of 0.5 IU/ml has therefore been adopted as an adequate titre. In our study, all mice developed a sufficient VNA titre against RABV after vaccination, but mice that had VNA titres of 2–64 IU/ml, which is higher than the accepted threshold, succumbed after challenge with FI-EBLV-2 virus, whereas all mice survived the challenge with FIRD-RABV. Due to this discrepancy between the VNA titres and cross-protection from the disease, people and animals need a post-exposure vaccination, regardless of their prior pre-exposure vaccination and antibody measurement result, if they are exposed to lyssaviruses. The intracranial route of infection was used because of the guidelines in the European Pharmacopoeia’s protocol. Pharmacopoeial methods are reference methods and the suitability of the method has been demonstrated. However, it should be noted that the intracranial exposure route used in our study is not the natural infection route.

Possible exposure to other lyssaviruses than RABV has especially raised concerns over whether RABV-based vaccines offer sufficient cross-protection and whether a higher cut-off for protection against EBLV-2 would be required. Studies have suggested that either higher serum VNA titres [34] or higher doses of rabies immunoglobulins are required [45] in humans. There is marked individual variation in the comparative neutralisation patterns of human sera against different lyssaviruses [34, 42]. Our studies indicated that the RABV antibody level does not clearly correlate with protection against EBLV-2. Therefore, laboratory personnel working with EBLV-2 should be regularly vaccinated and their antibody level should be monitored every 6 months, but it is still unclear what RABV antibody level would be protective. Therefore, booster vaccinations are recommended after possible exposure to lyssaviruses, and particular attention should be paid to the wound care after exposure. No laboratory accidents with other lyssaviruses than RABV have so far been reported.

Several factors influence the antibody level reached in an animal after vaccination, including the vaccine used, vaccine administration and the animal receiving the vaccine. Based on our studies, it is advisable that dogs that need a sufficient antibody test result due to international travel should be vaccinated twice with a rabies vaccine and then regularly receive a booster. Sihvonen et al. [40] demonstrated that a single vaccination of dogs with rabies vaccine induced moderate but short-term seroconversion in 96.9% of dogs, but in 17% of dogs the antibody titre did not last for a whole year in a population that had not previously been vaccinated. If vaccinations are only performed by veterinarians in veterinary clinics and vaccines are stored according to the manufacturer’s instructions, this might have a positive effect on the outcome. Factors such as the consistency (virus strain, cell culture and adjuvants) of the vaccine influence the vaccination response.

The difference between the proportions of dogs (around 11%) and cats (around 3%) that failed to reach the antibody level of 0.5 IU/ml might be caused by differences in genetic variation within the two species, as pedigree dogs are purebred, whereas the majority of cats in our study material were mixed breeds. The key genetic elements of immune responsiveness lie within the genes of the major histocompatibility complex (MHC). It has been suspected that cats have more limited diversity in immune response genes than dogs, but recent research has demonstrated similar variation in the MHC in cats to that found in dogs [51]. In our study, there was a significant difference in the ability of different vaccines to induce the antibody level of 0.5 IU/ml in dogs. This has also been reported in earlier studies [36–39], and may be due to differences in the immunogenicity of the vaccines or the potency of the vaccine batches. Moreover, veterinarians should note that there are significant differences between vaccines in their ability to induce an immune response. This difference between the vaccines was not statistically significant in cats, perhaps due to the type two error of an insufficient sample size, since there were so few (n = 25) failures to reach the threshold of 0.5 IU/ml in cats.

The time between vaccination and sampling was a significant risk factor for both dogs and cats failing to reach the threshold of 0.5 IU/ml. The antibody level peaks at slightly different times after vaccination with different vaccines, and the level starts to decline afterwards [37]. Larger dogs had a greater risk of failing to reach the required antibody level. Increasing the dose would not probably be a solution, since it has been shown that if there is a sufficient antigen to create a response, larger doses will not increase antibody production. Kennedy et al. [37] suggested that larger breeds might have deeper sub-cutaneous fat, which could reduce the level of the antibody response. The breed might also be a factor, not just the size of the dog, since even though most failures to reach the anticipated antibody level occurred in larger breeds, some smaller breeds had significant test failure rates [52]. Specific dog breeds have a genetically determined immune function, and a study has confirmed breed-specific serological response patterns to vaccination. The high interbreed and relatively low intrabreed variation in MHC alleles and haplotypes could provide an explanation for the reports of interbreed variation in immune responses to vaccines [53]. The higher risk of failing to reach 0.5 IU/ml for dogs and cats aged up to 1 year old could be due to the administration of vaccines before the animals had reached full immunocompetency, or due to maternal antibodies [36, 39].

In rabies-free countries, the rabies vaccination coverage of cats and dogs is likely to be lower than in rabies enzootic countries. Cats, especially in the countryside, often freely roam outside and are therefore able to encounter wildlife, consequently being at greater risk of exposure to rabid animals, and especially to bats, than dogs. Host switching of lyssaviruses from bats to mammals has successfully occurred in history [20, 21]. Natural spillover of EBLV-2 to other mammals than humans has not been demonstrated. Only a small proportion of cats failed to reach the required antibody level in our study, but the vaccination coverage of cats against rabies is assumed to be low in many rabies-free countries, at least in Finland. Based on the number of vaccination doses sold, we estimated that about 10–20% of 700,000 Finnish cats have been vaccinated against rabies. The rabies vaccination coverage is higher for dogs, estimated at around 40–65% based on the rabies vaccine sales in Finland.

We demonstrated in the mouse model challenge that an RABV-based animal vaccine offers cross-protection against challenge with the FI-EBLV-2 strain isolated from a bat. Therefore, it is recommended that even in rabies-free countries, dogs and cats that come into contact with lyssavirus and infected bats should be vaccinated against rabies. Booster vaccination is recommended for dogs, especially of large breeds. Moreover, veterinarians should pay attention to the choice of vaccine used.

## Conclusions

A veterinary RABV-based rabies vaccine, selected based on the ability to induce the best antibody response of the vaccines compared for dogs and cats in this study, offered cross-protection against EBLV-2 challenge in a mouse model in comparison with a challenge with RABV. Our mouse model provided an indication that the RABV antibody level and protection against EBLV-2 are not clearly correlated, and this most probably reflects the situation in other mammals and humans. There was a statistically significant difference in VNA titres between three groups of mice vaccinated with different vaccines or vaccination schemes. Approximately 11% of vaccinated dogs tested from the field failed to have an antibody titre of 0.5 IU/ml or more. By comparison, only a small proportion of cats failed to reach the required antibody level. Booster vaccination and vaccinating dogs and cats encountering bats is recommended, since vaccination also offers cross-protection against EBLV-2.
